# Evaluation of the Square Eyes Model as a Screening Tool for Identifying Digital Technologies in Wearable Camera Images Among Children: Laboratory Study

**DOI:** 10.2196/100559

**Published:** 2026-09-14

**Authors:** Charlotte Lund Rasmussen, Taren Sanders, Erin Kaye Howie, Amber Beynon, Danica Hendry, Juliana Zabatiero, Amity Campbell, Leon Straker

**Affiliations:** 1 School of Allied Health, Curtin University, Building 401, Kent Street, Perth, Western Australia, 6102, Australia, 61 (0) 8 92661771; 2 ARC Centre of Excellence for the Digital Child, Brisbane, Australia; 3 Institute for Positive Psychology and Education, Australian Catholic University, North Sydney, Queensland, Australia; 4 Department of Health, Human Performance and Recreation, University of Arkansas, Fayetteville, AR, United States

**Keywords:** children, wearable camera, machine learning, object recognition, image processing, screens

## Abstract

**Background:**

Accurate measurements of children’s digital technology use are essential for understanding its potential implications on health and well-being. Wearable cameras can provide such measurements, but image coding is a high burden for researchers. Machine learning–based object-recognition models have the potential to reduce this burden by identifying images containing technology.

**Objective:**

This study aims to evaluate the performance of an object recognition model, the Square Eyes model, as a screening tool for identifying technologies in wearable camera images among children for further human review, as well as to examine the potential influence of face-blurring methods on the model’s performance.

**Methods:**

This study used data collected on 48 children (aged 3‐14 y) during an approximately 1-hour laboratory session. The children performed various technology-related tasks while wearing a camera. A total of 221,226 images were coded by humans and processed through the Square Eyes model. The performance of the Square Eyes model as a screening tool was evaluated by (1) assessing agreement between the model and human coding; (2) evaluating the N-back algorithm, an algorithm embedded in the model aimed to flag images requiring human review; and (3) examining the potential influence of facial-blurring on model performance.

**Results:**

Humans detected technology in 92,745 (41.9%) images, and the Square Eyes model detected technologies with an overall accuracy of 78.0%. When considering specific technologies, agreement between the model and human coders was the highest for *Television* (n=19,148, 54.3%) and *Laptop* (n=8492, 44.5%) and lowest for smaller devices such as *Smartphone* (n=2600, 31.3%) and *Tablet* (n=3685, 25.1%). The model’s N-back algorithm effectively flagged images that required further human review, with only 7144 (3.2%) images that were not flagged for screening containing a human-coded technology. An explorative analysis indicated that using a square face-blurring with border could have reduced the model’s ability to accurately detect technologies.

**Conclusions:**

The Square Eyes model demonstrated overall satisfying accuracy in detecting technologies and successfully flagged images that required further review by humans. These findings suggest that the model could be used as an effective screening tool for reducing the burden of human coding. However, the model could be improved to more accurately detect smaller devices, and the form of facial blurring in images should be considered.

## Introduction

Children’s exposure to digital technologies has significantly increased over the last few decades, and the age of first exposure has decreased [1-3]. Along with the increase in daily exposure, there has also been a rapid expansion in the range of screen-based technology devices available to children, including both stationary (eg, television and desktop computers) and portable devices (eg, tablets and smartphones) [2]. Accordingly, the potential impacts of technology on children’s health and well-being are increasingly being debated and investigated [4]. Some studies have found excessive screen time to be associated with negative health effects such as obesity, sleep disturbances, and attention issues [5,6]. Other studies have reported positive associations between technology used for educational or social purposes and children’s learning capacities and social interactions [6-8]. Thus, the potential impacts of screen-based technology, in particular, on children’s health and well-being are complex and influenced not only by exposure time but also by the content and context of use [8,9]. Accurate measurements of technology use are therefore essential for a comprehensive understanding of the topic.

Children’s technology use has commonly been measured using parent- or self-reported measures such as questionnaires, diaries, or interviews [10]. Advantages of these methods include low cost and participant burden. However, data obtained from these methods can be inaccurate due to social desirability and recall biases [10,11]. Objective methods have the potential to overcome these limitations and accurately capture how and when children use technology. Wearable cameras are increasingly being used within health behavior research, given the ability to capture recordings of the wearer’s surroundings [12]. Wearable cameras are small devices typically affixed to clothing that can capture audio-visual recordings from a first-person perspective and have been used to investigate children’s and adolescents’ diets [13], exposure to food [14] and alcohol marketing [15], smoking [16], and travel to school [17]. Moreover, wearable cameras have been used to capture exposure, content, and context of technology use among children [18-20] and adolescents [21,22]. These findings demonstrate the potential of wearable cameras to provide objective and useful assessments of technology use.

Although wearable cameras have the potential to offer high-quality data on children’s technology use, there are challenges for researchers to consider. The manual coding of the data, the typical gold standard of processing, is very time-consuming and thus results in high researcher burden [10]. Work has been done to reduce this burden and improve interrater reliability between coders by developing a standardized protocol for annotating technology use among children based on wearable camera images [23]. Another option could be to use machine learning–based object-recognition methods, which is a subfield of computer vision that focuses on detecting, analyzing and classifying specific objects within images and videos [24]. Object recognition has been successfully implemented in several research areas, such as medical image analysis and security surveillance, and could significantly reduce the extensive researcher burden and potential errors associated with manual coding of images [24]. However, understanding the feasibility and validity of object recognition methods for identifying technologies in images from children wearing cameras is important for confidence in using this approach.

The accuracy of using object recognition methods for detecting technologies in wearable camera images is increasingly being investigated. One experimental study used an object recognition model to detect laptop, phone, and television screens on 1029 wearable camera images collected among adults and found a mean accuracy of 82% [25]. Another study used an object recognition model to identify screen-based technologies from wearable camera images collected from 5 adults over a 7-day period (n=30,661 images) [26]. Based on a subset of manually annotated images (n=2024), the model showed a mean accuracy of 85% for capturing phone, laptop, tablet, desktop computer, and television screens. Finally, one study used an object recognition model to identify technologies among 1800 images captured from 30 children aged between 3 and 5 years, who wore a camera over 2 days [19]. The authors found that the model could detect computers, smartphones, and televisions with a mean accuracy of 93%. Taken together, these findings support the feasibility of using object recognition models to identify technologies in wearable camera images with good accuracy.

However, there are several limitations of object recognition models. One limitation of such models is the limited ability to capture content and contextual aspects of technology use. Therefore, a hybrid approach that uses both the capacity of machine learning to identify images with technologies and the capacity of humans to code a range of aspects of technology use could together provide a more efficient and accurate data processing approach. The Square Eyes model was developed with this purpose in mind [27]. Specifically, this model was developed as a screening tool to identify the presence of technologies in wearable camera images and flag images that were likely to contain technologies and thus require further human review. The Square Eyes model is currently being used in the KidVision project [28] but has not been evaluated on datasets that were not used for model development. However, evaluating model performance on independent datasets is essential to determine generalizability and robustness.

Another challenge with the use of wearable cameras is ethical concerns, including data confidentiality and security, the intrusive nature of the device, and the risk of unintentionally recording sensitive images of participants and third parties [12]. These issues may be even more pronounced in studies with vulnerable populations such as children. An ethical framework has been developed by Kelly et al [29], which details these ethical issues and provides a guideline to ensure the protection of participants and third parties when using wearable cameras [29]. One of the recommendations is to obscure faces and identifying features, commonly achieved through image blurring techniques [29]. While this approach can alleviate some ethical concerns, a potential consequence is reduced image quality, which in turn may influence the performance of object recognition models [30]. However, the potential impact of face blurring on the performance of a model for identifying technologies is unknown.

The primary aim of this study was to evaluate the performance of the Square Eyes model as a screening tool for identifying technologies in wearable camera images among children for further review by humans. Additionally, the potential influence of face-blurring methods on the model’s ability to detect technologies was examined.

## Methods

### Study Design

This study was based on data collected in a laboratory-based setting while children performed a variety of tasks with and without technology.

### Ethical Considerations

The study gained ethical approval from the Curtin University Human Ethics Committee (HRE2022-0157). Written informed consent was obtained from caregivers and age-appropriate assent (written or verbal) was obtained from children.

### Participants

Children with typical development aged 3 to 14 years were recruited through university networks, word of mouth, flyers, and social media posts. Children with parent-reported psychological or physical clinical diagnoses that could affect their ability to follow instructions or perform tasks were excluded.

Recruitment was sex- and age-stratified for balanced representation across girls and boys and the following three age brackets: aged 3 to 5 years to represent a preschool population, aged 6 to 10 years to represent a primary school population, and aged 11 to 14 years to represent secondary school or early adolescent population. Age categories were defined to reflect transitions at school leading to different day-to-day structures in their environment [31,32] and acknowledge technology engagement differences across childhood [33,34].

### Data Collection

Data were collected between July 2022 and October 2022. Children attended a single approximately 1-hour data collection session at a Curtin University laboratory with their caregivers either alone (n=10) or with a friend or sibling (n=38). At each data collection session, 2 to 3 researchers with experience working with children were present. The laboratory was set up with different activities aimed at capturing different tasks with and without technology. Caregivers provided informed consent and completed a brief sociodemographic questionnaire. Depending on their age, children were then asked for verbal or written assent and subsequently fitted with the wearable camera.

One Brinno TLC120 Automated Wearable Camera operating at a 1-second frequency was fitted to the child’s chest using an adjustable chest-mounted harness. The Brinno camera weighs 101 g, is 60×60×35 mm in size, captures a 112° field of view. The Brinno camera was used to capture technology exposure during the data collection session and has previously been used in adolescent [21,22] and adult populations [35].

After becoming familiar with the laboratory and equipment, children performed a range of tasks for 2 to 3 minutes each. Tasks were generally completed in a standard order, but adjustments were made to maintain participant enjoyment. Participants engaged in tasks such as watching shows on a television or laptop, gaming, reading, drawing, and using a smartphone for taking photographs. Children received specific instructions for the postures or movements required for each task, allowing the Brinno camera to capture images from various positions and movement patterns. Table S1 in Multimedia Appendix 1 provides a full overview of the tasks completed by the children and the corresponding technological devices.

### Data Processing and Coding

#### Wearable Camera Images

The Brinno camera automatically generated time-lapse video files (.avi), which were subsequently downloaded and converted into individual images (.jpg) using the open-source software FFmpeg (version 4.3). The images were coded for technologies by a member of the research team, as well as being processed through the Square Eyes model. All images used for the primary analysis had faces blurred by a square with a border applied to the faces within the images using a custom-made Python program.

#### Human Coding

A member of the research team viewed images for each participant and recorded the corresponding technology code for each image in a spreadsheet alongside the image number (see Table 1 for an overview of technology codes). The human coder completed several hours of structured training using a study coding protocol prior to commencing annotation. Moreover, coding for the first 3 participants was reviewed by an experienced researcher, and feedback was provided before the remaining images were coded. Images were date- and time-stamped. Moreover, images were coded in chronological order. The coding protocol applied was as follows. First, the coder would assess if an image was suitable for coding. Images that were deemed uncodable due to poor image quality, such as motion blur, poor lighting, or being obscured were excluded. Next, for codable images, the coder would determine the presence of any visible technology. If no technology was identified, the image was classified as “no digital technology.” When digital technology was both visible and had its screen turned on, it was classified as “Technology 1,” referencing the technology associated with the primary task being performed by the child. In instances where multiple technologies appeared in the same image, additional devices were labeled as “Technology 2” or “Technology 3,” allowing up to 3 distinct technologies to be recorded per image.

**Table 1.** Technology codes and their definition used by the human coders and the Square Eyes model, respectively.

Code comparison number	Human coding		Square Eyes model	
	Technology code	Definition	Technology code	Definition
1	Desktop computer	A computer that fits on a desk but is not easily moved from place to place. Has a monitor, keyboard, mouse, and tower.	Desktop computer	A computer that fits on a desk but is not easily moved from place to place. Has a monitor, keyboard, mouse, and tower. Refers to the physical computer box rather than the monitor.
2	No matching code	—^a^	Computer monitor	The monitor of a computer that fits on a desk but is not easily moved from place to place.
3	No matching code	—	Computer mouse	The mouse of a computer that fits on a desk but is not easily moved from place to place.
4	No matching code	—	Computer keyboard	The keyboard of a computer that fits on a desk but is not easily moved from place to place.
5	Laptop computer	A computer that is small enough to be carried around easily and is flat when closed. Indicated by an inbuilt keyboard.	Laptop computer	A computer that is battery operated and has an integrated screen. Indicated by an inbuilt keyboard.
6	Smartphone	A handheld device that can be used as a small computer, connect to the internet, and run applications.	Mobile phone	A portable, handheld wireless device that enables voice calls, text messaging, internet access, and runs applications.
7	No matching code	—	iPod	A small electronic device for playing and storing digital audio and video files.
8	Tablet	A small, flat computer that is controlled by touching the screen with one’s finger or a special pen. Does not require a keyboard or mouse. Includes e-readers.	Tablet	A small, flat computer that is controlled by touching the screen with one’s finger or a special pen. Does not require a keyboard or mouse. Includes e-readers.
9	Television	A device shaped like a box or rectangle with a screen that receives electrical signals and changes them into moving images. Can stand alone or be mounted to a wall. Can be a smart TV (ie, internet connected)	Television	A device shaped like a box or rectangle with a screen that receives electrical signals and changes them into moving images. Can stand alone or be mounted to a wall.
10	No matching code	—	Remote	A handheld, portable device used to operate machinery, appliances, or digital equipment from a distance.
11	Handheld game console	Portable, self-contained devices that have a built-in screen, game controls, and speakers.	Handheld game console	Portable, self-contained devices that have a built-in screen, game controls, and speakers.
12	Combined controller	When the child has the Nintendo Switch controller which is controlled by 2 hands (ie, it has both the red and the blue parts together or the black controller in their hand).	No matching code	—
13	Single controller	When the child has the controller separated and/or in separate hands.	No matching code	—
14	Smartwatch	A watch that has an electronic screen with features of a smartphone or a computer. Includes fitness trackers, such as a FitBit.	No matching code	—
15	Uncodable: blurry	Any image or set of images where the image quality is so poor due to being blurred that the coder is unable to confidently determine what is occurring in all aspects of the image.	No matching code	—
16	Uncodable: obscured images	Any image or set of images where the image quality is so poor due to lighting that the coder is unable to confidently determine what is occurring in all aspects of the image. Poor lighting can include images that are too dark or too overexposed to accurately determine anything.	No matching code	—
17	Uncodable: poor lighting	Images that are completely black or fully blocked by something and cannot be coded as having an active screen-based media device according to the coding rules. Includes completely blacked images.	No matching code	—
18	Unsure	Coder is uncertain what is in the image.	No matching code	
19	No digital technology	No digital technology is visible (or active for room video).	No matching code	

a Not applicable.

A subset of the images from 3 participants (one from each age-group, n=12,024) were repeat coded by a second researcher, and interrater reliability was determined via assessing percentage agreement and Cohen κ [36]. Specifically, agreement in all coding options (n=14) for the “Technology 1” category was evaluated. Across all category codes and age-groups, very good interrater reliability was observed, with an average percentage agreement of 92.5% (SD 2.2%, range: 90.3%‐94.7%) and average κ of 0.88 (SD 0.03, range: 0.86‐0.92).

#### The Square Eyes Model

The Square Eyes model is a computer vision model developed to detect technologies in images captured from wearable cameras [27]. The model is a fine-tuning of the YOLO-v8 model developed by Ultralytics [37], which builds on the original YOLO architecture [38]. Specifically, the model was fine-tuned using a mix of wearable camera data and publicly available images of screen-based technologies to detect the 11 technology categories shown in Table 1. When making predictions, the Square Eyes model first removes overlapping duplicate detections using nonmaximum suppression. The remaining detections are then ordered by confidence score. The highest-confidence detection is assigned to “Device 1” and the second highest to “Device 2,” with further detections disregarded. For the purposes of visual review, bounding boxes are overlaid on the images based on the coordinates predicted by the model for each detection, indicating the estimated location and size of the detected device in the image.

The primary aim of the Square Eyes model was to minimize the burden on researchers to code images collected in free-living settings. In general, most images captured in free-living settings do not contain technologies and therefore do not need to be coded, but identifying which images do require coding is time consuming. To minimize false negatives from the model detection, the Square Eyes model is complemented by an N-back algorithm which flags images that are likely to contain a screen-based technology and requiring humans to review. The rationale for this algorithm was that the Square Eyes model was expected to miss some technologies due to, for example, motion blur and odd viewing angles. If a technology is present in 1 image, it is more likely to be present in adjacent images. Thus, the N-back algorithm flags an image as needing human review if it contains a technology or if it is within N chronologically adjacent images of an image that contains a technology. For example, if “n=2” and image 10 is identified as containing a technology, images number 8, 9, 10, 11, and 12 would be flagged for human review. Images where the model does not detect a technology or is not located within N frames of an image with model-detected technology are not flagged for human review. In the following, the term “detected” will be used when the Square Eyes model detected a technology on an image either as Device 1 or Device 2. The term “flagged” will be used to describe images identified by the N-back algorithm, which always include the “detected” images.

### Data Analysis

#### Statistical Approach and Model Evaluation

Basic descriptive statistics were used to describe the study sample and the distribution of technologies coded by humans and detected by the Square Eyes model, respectively.

The performance of the Square Eyes model as a screening tool was evaluated by investigating (1) agreement between the model and human coders for technology detection, (2) evaluation of the N-back algorithm for image flagging, and (3) examination of the potential influence of image blurring on model performance. Each analytical step is described in detail below.

#### Evaluation of Technology Detection by the Square Eyes Model

To evaluate the overall performance of the Square Eyes model as a screening tool for detecting the presence of any technology in all images, agreement between the model and human coding was assessed by calculating the number of true positives, false positives, true negatives, and false negatives using human coding as the reference standard. True positives were defined as images where both the model and human coders detected a technology; false positives were defined as images where only the model detected a technology; true negatives were defined as images where neither the model nor the human coders detected a technology; and false negatives were defined as images where only the humans detected a technology. Moreover, sensitivity (true positives / (true positives + false negatives)), specificity (true negatives / (true negatives + false positives)), and overall accuracy ((true positives + true negatives) / (true positives + true negatives + false positives + false negatives)) were calculated.

We further evaluated the Square Eyes model performance in detecting different technology types by assessing agreement between model detection and human coding separately for each technology category. To enable this comparison, a 6-category variable (“any technology combination”) was created based on the presence of specific technologies identified by either the model or human coders. Specifically, this was a nonexclusive variable where each category described when a technology was either detected alone or in combination with another technology. For example, the *Any television* category included images where a television was detected by the model (as Device 1 or Device 2) and/or coded by humans (as Technology 1, 2, or 3), regardless of whether other technologies were also present. This resulted in the following categories: *Any computer, Any gaming, Any laptop, Any smartphone, Any tablet,* and *Any television*. Table S2 in Multimedia Appendix 2 provides an overview of how technology codes used by the Square Eyes model and the human coders were matched to create the “any technology combination” variable. Agreement between the model and human coding was then evaluated separately for each technology category. Percent agreement between the human coders and the Square Eyes model, and prevalence-adjusted bias-adjusted kappa (PABAK) were calculated separately for each category [36,39]. The PABAK was calculated to account for the potential influence of imbalanced prevalence of some technology types on agreement estimates. The PABAK values were interpreted using the Landis and Koch [40] classification scale as below chance agreement <0.00, slight agreement 0.00‐0.20, fair agreement 0.21‐0.40, moderate agreement 0.41‐0.60, substantial agreement 0.61‐0.80, and almost perfect agreement 0.81‐1.00. Of note, negative PABAK values are more likely to arise in situations with substantial class imbalance and high true negative frequency. In this context, it is encouraged to interpret the PABAK values alongside category-specific percent agreement as the negative values may reflect limited sensitivity for infrequent categories rather than systematic disagreement [36,39].

#### Evaluation of Image Flagging by the Square Eyes Model With the N-Back Algorithm

To enable evaluation of the N-back algorithm, the number of images flagged and not flagged for human review when “n=2” was quantified. The frequency and prorata proportion of technology types were calculated among images that were not flagged for human review, but where humans had coded the presence of a technology. This was done to identify which technologies were most likely to be overlooked by the N-back algorithm. The performance of the N-Back algorithm was further evaluated by calculating sensitivity, specificity, and the proportion of images flagged for human review across *N* values ranging from 0 to 10. These metrics were used to investigate the expected trade-off between technology flagging performance and human coding burden as the buffer size (*N*) increased.

#### Examination of the Influence of Image Blurring on Technology Detection by the Square Eyes Model

An explorative assessment was conducted to examine if the Square Eyes model performed differently on images in which faces had been blurred compared to images where faces had not been blurred (ie, nonblurred images) or were blurred in a different manner. For this assessment, images from a single participant were examined to identify discrepancies in technology detection attributable to the blurring process. The blur was originally applied as a square with a border, which may resemble a digital screen. Thus, to explore whether the detection was driven by the presence of the blur border or the shape of the blur, alternative blur styles were tested—specifically, a square blur without a border and a circular blur without a border.

## Results

### Participants and Coding of Images

Among the 48 included participants, 21 were boys and 27 were girls (overall mean age 8.5, SD 3.3 y; boys: mean age 8.2, SD 3.2 y; girls: mean age 8.7, SD 3.5 y). A total of 221,226 images were coded by humans and processed through the Square Eyes model.

Humans detected technology in 92,745 (41.9%) images, of which *Television* (n=40,231; 43.4%) and *Laptop* (n=12,717; 13.7%) were the most frequently coded as Technology 1 (Table S3 in Multimedia Appendix 3). The Square Eyes model detected technology in 101,075 (45.7%) images, with *Television* (n=23,590; 23.3%) and *Computer monitor* (n=17,766; 17.6%) being the most commonly detected as Device 1 (Table S4 in Multimedia Appendix 3).

### Evaluation of Technology Detection by the Square Eyes Model

Both humans and the Square Eyes model detected a technology in 72,470 images (ie, true positive rate=32.8%) and no technology in 99,876 images (ie, true negative rate=45.1%). A technology was detected by humans only in 20,275 images (ie, false negative rate=9.2%) and by the Square Eyes model only in 28,605 images (ie, false positive rate=12.9%). Thus, overall sensitivity was 78.1%, specificity was 77.7%, and overall accuracy for detecting technologies was 78.0%.

Table 2 shows the frequency distribution of the 6 “any technology combination” categories for images where both the humans and the Square Eyes model had detected a technology (n=74,470). The most frequently coded technologies based on the human coding were *Any television* (n=32,662) and *Any laptop* (n=17,880). The Square Eyes model most frequently detected *Any television* (n=21,762) and *Any computer* (n=34,530). Overall, there was a below chance agreement between the human coding and the model. Mean percentage agreement was 32.8% (14.0%‐54.3%) and the PABAK score ranged between –0.719 and 0.086. The highest level of agreement was observed for *Any television* (n=19,148, 54.3%; PABAK=0.086), while the lowest level of agreement was found for *Any gaming* (n=1013, 14.0%; PABAK=−0.719).

**Table 2.** Frequency and agreement of “any technology combination“ based on human coding and Square Eyes model detection.

Technology	Human coding, n	Square Eyes model, n	Total N^*a^	Agreement, n (%)	PABAK^b^
Any computer	9989	34,530	34,908	9611 (27.5)	−0.449
Any gaming	6911	1320	7218	1013 (14.0)	−0.719
Any laptop	17,880	9703	19,091	8492 (44.5)	−0.110
Any smartphone	5346	5567	8313	2600 (31.3)	−0.374
Any tablet	6419	11,932	14,666	3685 (25.1)	−0.497
Any television	32,662	21,762	35,276	19,148 (54.3)	0.086

a Total N* coded by either by humans and/or detected by the model.

b PABAK: prevalence-adjusted bias-adjusted kappa. Frequency and PABAK calculations based on images with both human coding and the model detecting a technology (N=74,470).

### Evaluation of Image Flagging by the Square Eyes Model With N-Back Algorithm

The N-back algorithm (with n=2) flagged 148,546 (67.2%) images that would require human review and 72,680 (32.8%) images were not flagged.

Among the flagged images, a technology was coded by humans only in 13,131 (8.8%) of the flagged images, and a technology was detected by the model only in 28,605 (19.3%) images. Neither the humans nor the model detected a technology in 34,340 (23.1%) of the flagged images. In 72,470 (48.8%) of the flagged images, both humans and the model detected a technology. When considering the images not flagged for human review, 7144 (9.8%) images had a technology coded by humans only, while 63,536 (90.2%) images did not have a technology coded by humans. None of the images had a technology detected by the model only.

Table 3 shows the proportion of human-coded technologies (based on Technology 1) that were not flagged for human review by the N-back algorithm. Among the images that were not flagged, the most frequently missed technologies (based on prorata percentages) were *Smartwatch* (n=115, 46.7%), *Single controller* (n=62, 19.8%), and *Tablet* (n=1518, 15.1%).

**Table 3.** Proportion of human-coded technologies on images not flagged for review by the N-back algorithm.

Technology 1 (human-coded)	Total images, n	Nonflagged images, n (%)
Combined controller	3675	157 (4.3)
Desktop	10,714	363 (3.4)
Handheld gaming	7678	146 (1.9)
Laptop	12,527	978 (7.8)
Single controller	313	62 (19.8)
Smartphone	6668	458 (6.9)
Smartwatch	246	115 (46.7)
Tablet	10,066	1518 (15.1)
Television	39,145	3346 (8.5)

**Figure 1.**
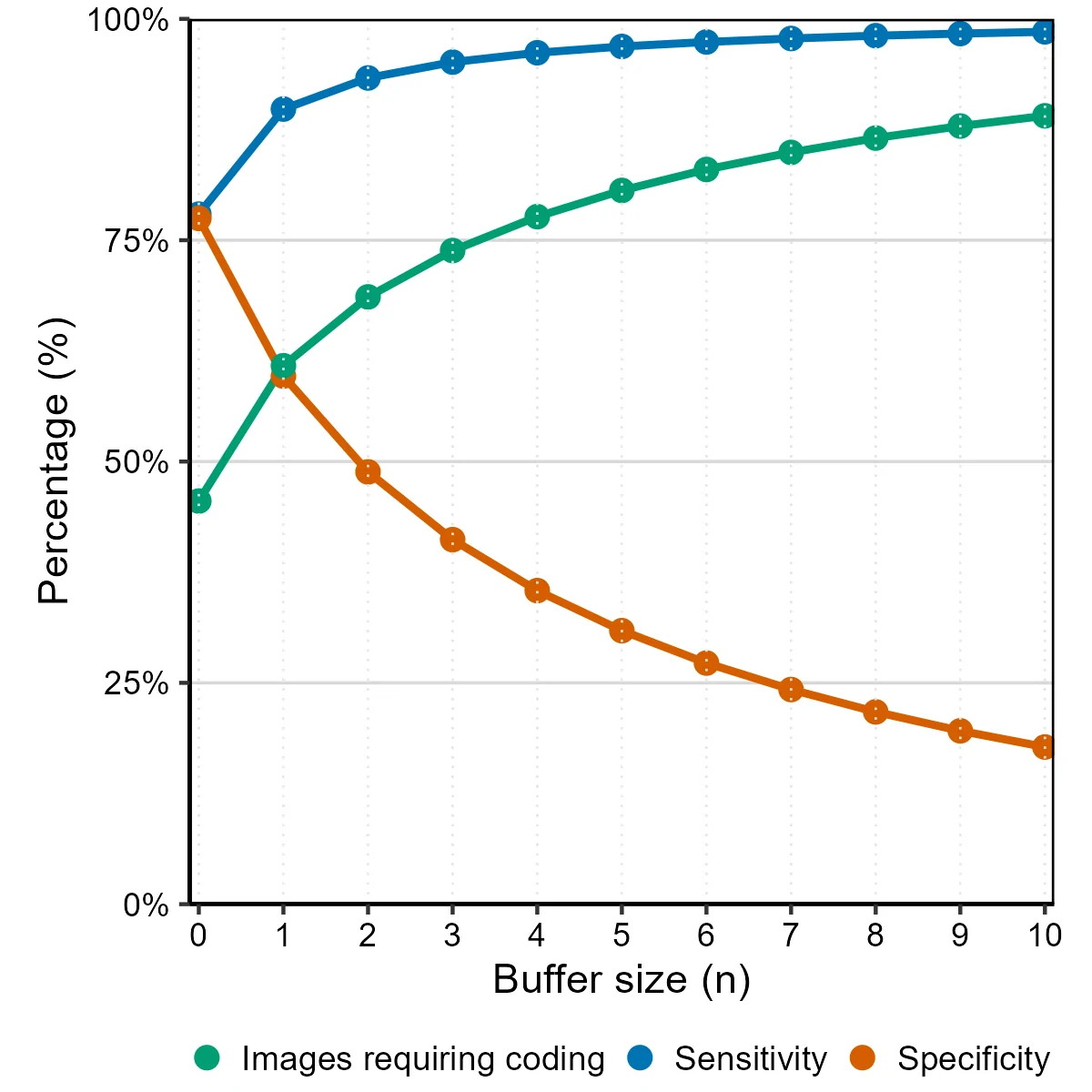
Effect of N-back buffer size on sensitivity, specificity, and images flagged for human review.

Figure 1 illustrates the trade-off between sensitivity, specificity, and the proportion of images flagged by the N-back algorithm as the N-back buffer size increases. Specifically, the figure illustrates that as the N-back buffer size increases, sensitivity increases, and the proportion of images flagged for human review increases while specificity decreases.

### Influence of Face Blurring on Technology Detection by the Square Eyes Model

Detection performance by the Square Eyes model on blurred and nonblurred versions of 5667 images from a single participant was examined. The model detected technology on slightly more blurred images than on their nonblurred counterparts. Specifically, the model detected a technology on 173 blurred images, which was not detected when the images were nonblurred. In contrast, 60 images were classified as having a technology when nonblurred but not when they were blurred.

**Figure 2.**
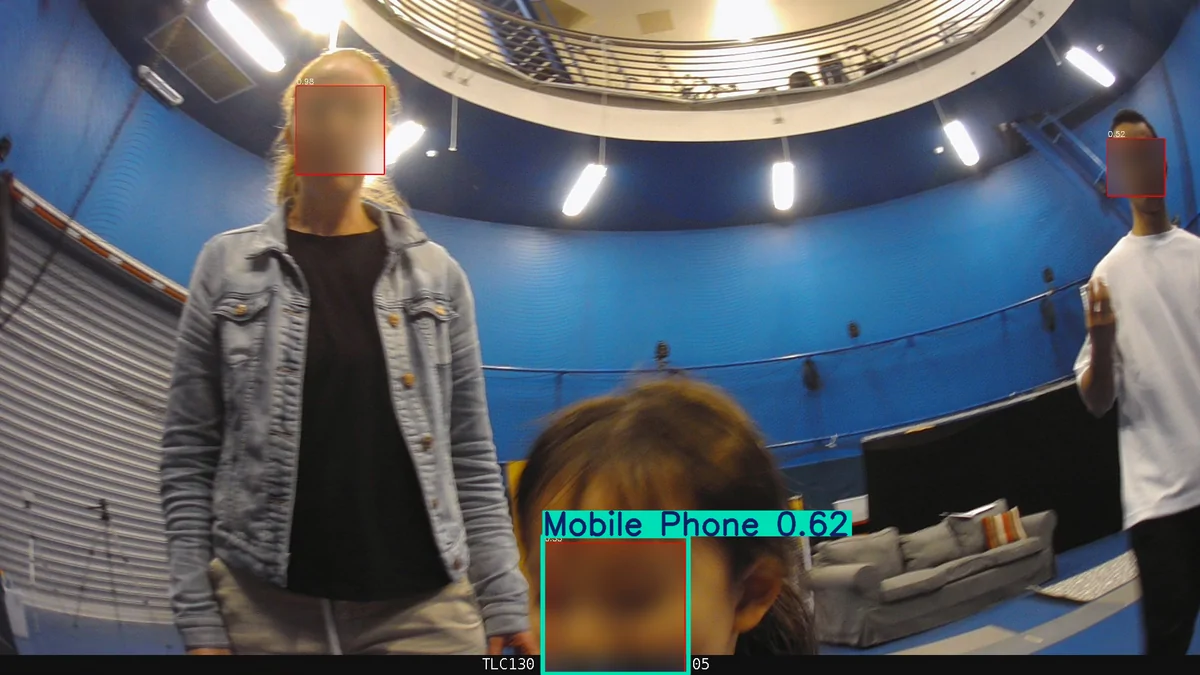
Example case of the Square Eyes model detecting a face blur as technology.

**Figure 3.**
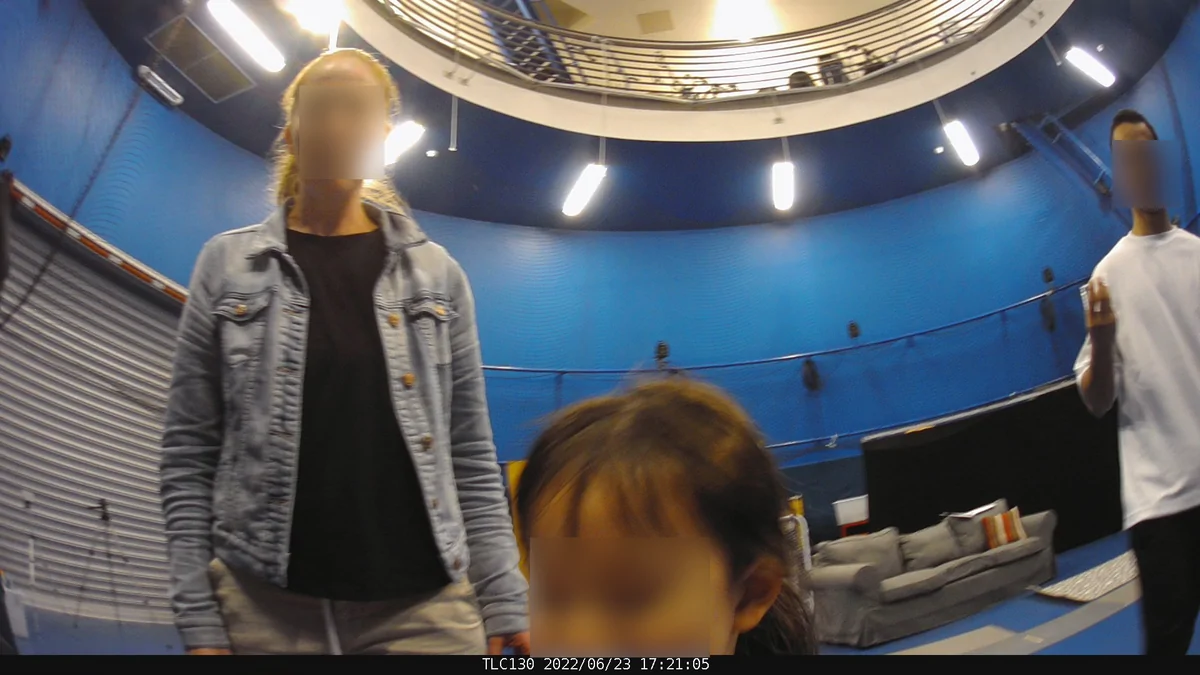
Example case of alternative face blur (square shape without border).

**Figure 4.**
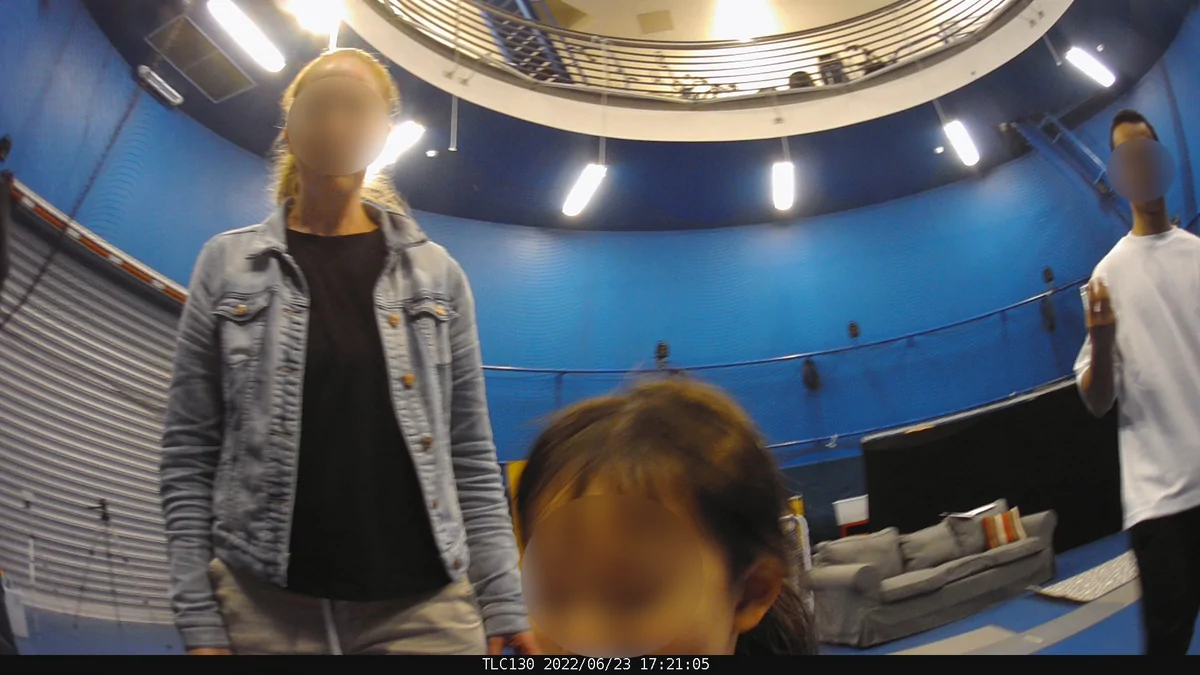
Example case of alternative face blur (circular shape).

Figure 2 represents an example where the model detected a technology only in the blurred version. The bounding box for the model predictions is shown as a blue square, illustrating that the model detected a *Mobile phone*. Figures 3 and 4 show examples of the alternative blur styles. When the blur was applied either as a square without border (Figure 3) or in a circular shape without border (Figure 4), the model no longer detected technologies in these images. Thus, this suggests that the specific characteristics of the original blur effect influenced the model’s misclassification rather than the presence of blurring itself.

## Discussion

### Overall Summary

This study aimed to evaluate the performance of the Square Eyes model as a screening tool for identifying technologies in wearable camera images among children for further review by humans. The Square Eyes model demonstrated good agreement with human coders in detecting technologies with an overall accuracy of 78.0% and a false negative rate of only 9.2%. However, when considering specific technologies, lower levels of accuracy were found for some devices (eg, *Smartphone* and *Computer*). The model’s N-back algorithm effectively flagged images that required further human review. Specifically, of the complete sample of 221,226 images, only 7144 were not flagged for screening and contained a human-coded technology, thereby reducing the false negative rate from 9.2% to 3.2%. Our explorative examination of the potential influence of the image blurring method on the model performance revealed that using a square blurring with border could have reduced the model’s ability to accurately detect technologies.

### Overall Model Performance in Detecting Technologies

When assessing the Square Eyes model’s ability to detect technologies, it should be noted that the model was intended to be used as a screening tool for reducing researchers’ burden of manually annotating wearable camera images rather than as a replacement for human coding. Nevertheless, the model showed good agreement with human coding, with sensitivity of 78.1%, specificity of 77.7%, and overall accuracy of 78.0%. Thus, the model failed to detect a technology on 9.2% (n=20,275) of all images. Given that each image was taken at a 1-second frequency, this corresponds to approximately 338 minutes of missed technology exposure out of a total of 1546 minutes of technology exposure as identified by humans. Moreover, differences were observed in the performance for detecting specific technologies. We found that the best agreement between the Square Eyes model and human coders was for *Television* (54.3%, n=19,148) and *Laptop* (44.5%, n=8492). In contrast, lower levels of agreement were found for *Smartphone* (31.3%, n=2600), *Computer* (27.5%, n=9611), *Tablet* (25.1%, n=3685), and *Gaming devices* (14.0%, n=1013). Thus, a relatively high proportion of handheld devices was not identified by the model. This finding aligns with known limitations of object recognition models that tend to show lower accuracy in detecting smaller objects compared to larger objects as these are often partially occluded and with limited discriminative visual features [38]. The poorer performance for detecting *Computer* by the Square Eyes model may have been a result of overlap with related categories being present in the image (eg, *Laptop*, *Monitor*, or *Keyboard*), which complicates correct classification. The poor detection of particularly handheld devices should be acknowledged as a key limitation of using the Square Eyes model as a standalone tool given that such devices are commonly used among children and adolescents [41].

The observed accuracies of the Square Eyes model are generally lower than what has been reported in studies evaluating the use of similar object recognition models for identifying technologies in wearable camera images. One prior study based on 1029 images from an adult sample reported detection accuracies ranging between 81% and 84% for phones, laptops, and televisions [25]. Another adult-based study found a mean accuracy of 85% for identifying phones, laptops, tablets, desktops, computers, and television screens based on 215 images [26]. A study based on 1800 images from cameras worn by children aged 3 to 5 years observed a mean accuracy of 93% for identifying computers, smartphones, and televisions [19].

Several methodological factors may explain the lower accuracy of the Square Eyes model found in the present study. First, we evaluated the accuracy of the Square Eyes model based on an independent set of images. In contrast, in all the previous studies, model performance was evaluated based on a subset of images drawn from a sample used for training the models. While this approach is commonly used for model evaluation, it is likely that performance estimates were increased due to similarity between the training and testing datasets. Moreover, the subsets were substantially smaller (n=215‐1800 images) than the number of images used for evaluating the model in the current study (n=221,226). The smaller samples may have resulted in higher performance estimates as a result of issues such as overfitting [42]. The studies by Hou et al [19] and Li et al [25] excluded images with multiple screens in the same image, whereas multiple devices were present on the images used in the current study. We elected to keep multiple screens as this is more likely what will be found in free-living environments. However, the presence of multiple screens will increase the challenge of object recognition for the model, particularly when screens partially occlude one another. The studies by Li et al [25] and Su et al [26] were based on adult samples. It can be expected that images from cameras worn by adults are less influenced by motion blur than those from cameras worn by children who typically have more rapid movements than adults. Moreover, the lower height of children may influence the ability of wearable cameras to capture stationary technologies, such as televisions, which may be placed above the children’s eye line. Finally, low-quality images affected by motion blur were excluded prior to analysis in the child-based study by Hou et al [19], which likely improved model performance. Taken together, differences in training–testing dataset overlap, sample size, image complexity, and image quality likely contribute to the lower agreement observed in the present study.

### The Potential for Reducing Researcher Burden

The model’s N-back algorithm effectively excluded 32.8% (n=72,680) of the images from requiring manual annotation, indicating a substantial reduction in workload. Importantly, of the images not flagged for human review, 90.2% (n=63,536) did not contain a human-coded technology, suggesting that most images were correctly identified as not requiring human review. Across the images not flagged for human review but containing a human-coded technology (n=7144, 3.2%), these images primarily contained smaller devices (eg, smartwatches and smaller gaming devices). Thus, while the algorithm reduced human coding burden by approximately one-third, some smaller technologies were more frequently missed. Nevertheless, our findings suggest that the Square Eyes model could be used as an efficient tool for reducing human coding burden.

To our knowledge, no prior study has reported an evaluation of the ability of object recognition models to reduce overall researcher burden for processing wearable camera images. Decreasing reliance on intensive manual coding may contribute to greater research workforce sustainability by reducing a highly time-consuming task. Thus, we encourage future research to explore the use of similar hybrid approaches. Such approaches could facilitate the use of object recognition models to identify images likely to contain technologies, followed by human coders to confirm the presence of technologies. Moreover, human coding would remain essential for obtaining information on the content and context of technology use. Additionally, engaging with parents and children in study designs may further facilitate the inclusion of larger and more diverse study populations by supporting feasibility and acceptability of wearable camera research.

An additional consideration for future research relates to the rapid evolution of wearable camera devices, which include higher quality images and continuous video recording. The Square Eyes model was trained and developed for still-image object recognition and thus, we encourage future work to test the feasibility of model development for video-based analysis. This would likely involve further development of the current YOLOv8-based framework to incorporate temporal information across continuous frames and to optimize performance for small objects such as smartphones and handheld devices. Future advances in AI are likely to enhance model performance and should be evaluated as advanced models become available, building on the knowledge from this study.

### Potential Impact of Face Blurring

The primary purpose of obscuring faces using a blurring technique is to ensure third-party privacy [29]. However, a potential consequence may be reduced model performance [30]. In the current study, we observed that the use of a square-bordered blur was sometimes mistakenly identified as a technology by the Square Eyes model. In contrast, this did not appear to be the case when using either a nonbordered square or nonbordered circular face blur. Thus, the performance of the Square Eyes model for detecting technologies reported above may have been reduced by the facial blur technique applied to the images in this study. To our knowledge, the potential influence of facial blur techniques on the performance of object recognition models used for technology detection in wearable camera images has not been previously examined. However, our findings contradict those of a study assessing the effect of face blurring on the performance of a deep-learning model used for human pose estimation [43]. The authors concluded that face blurring had minimal impact on model performance when the model was trained using face-blurred images. However, this model was designed for whole-body posture recognition, not for the detection of technologies. As ensuring privacy is important in wearable camera research, we encourage future work to evaluate how different face-blurring techniques may influence the performance of machine learning models used for detecting technologies. This may involve training models directly on face-blurred images and comparing different blurring features (eg, circular vs square) across diverse real-world conditions to support the development of standardized ethical pipelines for analyzing wearable camera images.

### Strengths and Limitations

A strength of this study was the large data sample of images captured by wearable cameras worn by children aged 3 to 14 years. Few studies have evaluated the use of wearable cameras and machine learning models for technology identification among children, and thus our study substantially adds to an underinvestigated research area. Moreover, images captured among children are likely to have lower visual quality due to increased motion blur compared with images captured among adults. Thus, evaluating the Square Eyes model using a visually challenging dataset provided a rigorous test of model robustness. This was further supported by using images that were not part of the dataset used for training the model. Specifically, models often demonstrate reduced performance when applied to datasets from other samples and settings. Our results, therefore, support the external validity of the Square Eyes model. Nevertheless, some limitations to the dataset should be acknowledged. This study was based on images obtained in a laboratory, which limits generalizability to free-living settings. Specifically, in free-living settings, the quality of images obtained with wearable cameras is likely influenced by substantial variability in lighting, postures, and movements, and thus image quality. Moreover, patterns of technology use in a laboratory setting may not reflect real-world settings where children are likely to engage with multiple technologies across varied physical environments and social settings. Such factors are likely to reduce detection performance of object recognition models. Thus, the findings should be interpreted as a proof-of-concept under controlled conditions rather than estimates of real-world applicability. Given the high burden of human coding, only one coder was used. Although coder training and initial review procedures were implemented to ensure high-quality coding, reliability was not assessed across a larger subset of the dataset. Therefore, we cannot assume that the human-coded reference was free from misclassifications. The potential influence of the facial blurring technique on model performance was evaluated using a small sample of images from a single participant. This analysis was explorative, and it was beyond the scope of the study to estimate performance differences between facial blur methods for the whole sample. Therefore, given the limited sample size and lack of systematic comparison, these findings regarding the impact of facial blur methods should be interpreted with caution.

### Conclusions

We found that the Square Eyes model could detect technologies with overall satisfying accuracy and successfully flag images that required further review by humans. These findings suggest that the model can be used as an efficient screening tool to reduce human coding burden for researchers processing wearable camera images of children. However, the model could be improved for accurately detecting smaller devices, such as smartphones. Moreover, we found that the method used for facial blurring might influence model performance. We encourage further research to evaluate the use of similar hybrid approaches that use machine learning models to reduce human coding burden for both technology identification and other important aspects of technology use, such as context and content.

## Supplementary material

10.2196/100559 Multimedia Appendix 1 Overview of tasks completed by children during the data collection session.

10.2196/100559 Multimedia Appendix 2 Overview of matching between technology codes used by the human coders and the Square Eyes model.

10.2196/100559 Multimedia Appendix 3 Frequency of technologies as coded by humans and detected by the Square Eyes model.

## Abbreviations

PABAK: prevalence-adjusted bias-adjusted kappa
