# Supplementary material for: Evaluation of the Square Eyes Model as a Screening Tool for Identifying Digital Technologies in Wearable Camera Images Among Children: Laboratory Study
Source: J Med Internet Res. 2026 Sep 14;28:e100559. doi: 10.2196/100559 (PMC13574268; doi:10.2196/100559)
Supplement: Supplementary file 1 [file jmir-v28-e100559-s001.docx]

**Supplementary file A**

**Table A1:** *Tasks completed by children during the data collection session.*

| **Technology / non-technology** | **Position/movement** | **Details of task** |
| --- | --- | --- |
| Television | Sitting on bean bag | Watching a programme of child’s choice on television. |
| Television | Lying (prone) | Watching a programme of child’s choice on television. |
| Laptop | Lying (side lying) | Watching a programme of child’s choice via laptop. |
| Laptop | Sitting (chair) | Watching a programme of child’s choice via laptop. |
| Handheld game | Sitting (couch) | Playing Mario Kart via Nintendo Switch using just Switch screen. |
| Handheld game | Standing | Playing Mario Kart via Nintendo Switch using just Switch screen. |
| Book * | Lying (prone) | Reading a book. |
| Stationary game | Sitting (couch) | Playing Mario Kart via Nintendo Switch connected to television. |
| Magnetic Tiles*/ Jenga | Sitting (floor) | Playing with Magnetic tiles on floor. |
| Active game | Standing while moving/jumping | Playing Just Dance or NS Sports via Nintendo Switch connected to television. |
| Desktop | Sitting (chair) | Drawing or reading on desktop computer. |
| Tablet | Standing | Drawing using a tablet application. |
| Pencils/crayons* | Standing | Drawing and colouring in with paper and coloured pencils/crayons. |
| Tablet | Standing whilst moving | Participating in Cosmic Kids Yoga video, or similar age-appropriate movement based you-tube video. |
| Smartphone | Walking; Jumping; Running; Skipping | Taking selfies/pictures of posters whilst walking/jumping/running/skipping. |
| Ball* | Standing while throwing/ catching | Throwing/ catching a netball/ basketball. |
| Smartphone | Lying supine/ crook lie (floor) | Watching “Smiling Minds” Sea Creatures video or video of choice. |
| Smartwatch | Jumping; Handstands | Jumping and hand stands then checking heart rate/steps via smartwatch. |
| Smartwatch | Running; Cartwheels | Running and then checking heart rate/steps via smartwatch screen. |
| Smartwatch | Climbing stairs | Climbing stairs and then checking heart rate /steps via smartwatch screen. |

**Non-technology-based tasks.*
