# Supplementary material for: Evaluation of the Square Eyes Model as a Screening Tool for Identifying Digital Technologies in Wearable Camera Images Among Children: Laboratory Study
Source: J Med Internet Res. 2026 Sep 14;28:e100559. doi: 10.2196/100559 (PMC13574268; doi:10.2196/100559)
Supplement: Supplementary file 2 [file jmir-v28-e100559-s002.docx]

**Supplementary file B**

**Table B1.** M*atching between technology codes used by the human coders and the Square Eyes model were matched to create the ‘any technology combination’ variable.*

| **Human coding** | **Square Eyes model** | **Any technology combination** |
| --- | --- | --- |
| Desktop | Desktop computer; Computer monitor | Any computer |
| Handheld game console | Handheld game console | Any gaming |
| Laptop | Laptop computer | Any laptop |
| Smartphone | Mobile phone | Any smartphone |
| Tablet | Tablet | Any tablet |
| Television | Television | Any television |
