# Supplementary material for: Evaluation of the Square Eyes Model as a Screening Tool for Identifying Digital Technologies in Wearable Camera Images Among Children: Laboratory Study
Source: J Med Internet Res. 2026 Sep 14;28:e100559. doi: 10.2196/100559 (PMC13574268; doi:10.2196/100559)
Supplement: Supplementary file 3 [file jmir-v28-e100559-s003.docx]

**Supplementary File C**

**Table C1.** *Frequency of technologies coded by humans.*

| **Category** | **Technology 1**  **(n(%))** | **Technology 2 (n(%))** | **Technology 3 (n(%))** |
| --- | --- | --- | --- |
| Combined controller | 3,675 (4.0) | 2,889 (15.7) | 52 (7.0) |
| Desktop | 11,031 (11.9) | 61 (0.3) | 35 (4.7) |
| Handheld gaming | 7,692 (8.3) | 12 (0.1) | 0 (0) |
| Laptop | 12,717 (13.7) | 8,114 (44.1) | 83 (11.2) |
| Single controller | 313 (0.3) | 2,645 (14.4) | 390 (52.7) |
| Smartphone | 6,724 (7.3) | 307 (1.7) | 83 (11.2) |
| Smartwatch | 246 (0.3) | 40 (0.2) | 0 (0.0) |
| Tablet | 10,116 (10.9) | 1,741 (9.5) | 34 (4.6) |
| Television | 40,231 (43.4) | 2,577 (14.0) | 63 (8.5) |
|  |  |  |  |
| Total | 92,745 (100.0) | 18,386 (100.0) | 740 (100.0) |

**Table C2**. *Frequency of technologies detected by the Square Eyes Model.*

| **Category** | **Device 1**  **(n (%))** | **Device 2**  **(n (%))** |
| --- | --- | --- |
| Computer Keyboard | 9,231 (9.1) | 9,338 (29.0) |
| Computer Monitor | 17,766 (17.6) | 4,185 (13.0) |
| Computer Mouse | 8,250 (8.2) | 3,920 (12.0) |
| Desktop Computer | 1,567 (1.6) | 1,848 (5.7) |
| Handheld game console | 1,258 (1.2) | 138 (0.4) |
| iPod | 3,323 (3.3) | 1,431 (4.4) |
| Laptop Computer | 9,605 (9.5) | 940 (2.9) |
| Mobile Phone | 5,795 (5.7) | 1,116 (3.5) |
| Remote | 5,401 (5.3) | 3,028 (9.4) |
| Tablet | 15,289 (15.1) | 3,324 (10.3) |
| Television | 23,590 (23.3) | 2,951 (9.2) |
|  |  |  |
| Total | 101,075 (100.0) | 32,219 (100.0) |
